# Elevated tropospheric CO_2_ and O_3_ concentrations impair organic pollutant removal from grassland soil

**DOI:** 10.1038/s41598-018-23522-z

**Published:** 2018-04-03

**Authors:** Fuxun Ai, Nico Eisenhauer, Alexandre Jousset, Olaf Butenschoen, Rong Ji, Hongyan Guo

**Affiliations:** 1 Stake Key Laboratory of Pollution Control and Resource Reuse, School of Environment, Nanjing University, Nanjing, 210023 China; 2 German Centre for Integrative Biodiversity Research (iDiv) Halle-Jena-Leipzig, Deutscher Platz 5e, 04103 Leipzig, Germany; 3 Institute of Biology, Leipzig University, Deutscher Platz 5e, 04103 Leipzig, Germany; 4 Institute of Environmental Biology, Utrecht University, Padualaan 8, 3584 CH Utrecht, The Netherlands; 5 J.F. Blumenbach Institute of Zoology and Anthropology, University of Göttingen, Berliner Str. 28, 37073 Göttingen, Germany

## Abstract

The concentrations of tropospheric CO_2_ and O_3_ have been rising due to human activities. These rising concentrations may have strong impacts on soil functions as changes in plant physiology may lead to altered plant-soil interactions. Here, the effects of eCO_2_ and eO_3_ on the removal of polycyclic aromatic hydrocarbon (PAH) pollutants in grassland soil were studied. Both elevated CO_2_ and O_3_ concentrations decreased PAH removal with lowest removal rates at elevated CO_2_ and elevated O_3_ concentrations. This effect was linked to a shift in soil microbial community structure by structural equation modeling. Elevated CO_2_ and O_3_ concentrations reduced the abundance of gram-positive bacteria, which were tightly linked to soil enzyme production and PAH degradation. Although plant diversity did not buffer CO_2_ and O_3_ effects, certain soil microbial communities and functions were affected by plant communities, indicating the potential for longer-term phytoremediation approaches. Results of this study show that elevated CO_2_ and O_3_ concentrations may compromise the ability of soils to degrade organic pollutants. On the other hand, the present study also indicates that the targeted assembly of plant communities may be a promising tool to shape soil microbial communities for the degradation of organic pollutants in a changing world.

## Introduction

Global industrialization has led to an increase of tropospheric carbon dioxide (CO_2_) concentration from approximately 280 ppm in pre-industrial times to approximately 380 ppm nowadays, and it is expected to continue increasing in the future^1,2^. Alongside, the average surface ozone (O_3_) concentration has increased from an estimated pre-industrial value of 10 ppb to 20–45 ppb in the mid-latitudes of the northern hemisphere at a rate of 0.5–2% per year over the last decades^2–4^.

Industrialization has further led to a global pollution by organic pollutants, including polyaromatic hydrocarbons (PAHs)^5^. PAHs have mutagenic and carcinogenic properties, and show a high persistency in the environment^6,7^. Due to PAH contamination, huge areas are not suitable for agriculture or livestock anymore, and remediation of PAHs from soils is a priority goal to ensure food safety^8,9^. Among the proposed approaches, phytoremediation appears as an efficient and environment-friendly approach to remove PAHs from soils from large surfaces^10^. In most cases, phytoremediation of PAHs from soils was conducted by a single plant species^11,12^ and the mechanisms linking plants and PAH removal are still elusive. Moreover, it remains unclear whether and how global environmental change agents will affect phytoremediation of PAHs from soils.

In the present study, the potential effects of rising tropospheric O_3_ and CO_2_ concentrations on the natural ability of soils to degrade PAHs were investigated, and if such potential effects are altered by plant diversity. Soil microorganisms are important mediators of global change effects as several soil bacteria and fungi produce enzymes that break down PAHs, contributing to soil remediation. This effect is especially pronounced in microbial communities associated with plant roots^13,14^, where microbes are directly stimulated by the presence of labile plant-derived carbon. Since soil microbial communities and their activity are profoundly affected by plant community composition^15–18^, PAH removal may be influenced by changes in plant community composition and diversity. Plant diversity effects on soil biota may alter the functioning of soils, such as changes in the decomposition of organic matter^19,20^ and the sequestration of carbon in soil^18^. Increasing plant species richness may enhance the diversity of soil biota by combining different root morphology, root chemical composition, and temporal variability of resource inputs^18,21^. In addition, the functional composition of plant communities can drive belowground communities and processes^22,23^, *e.g*. through specific plant traits affecting nutrient availability^24–26^.

Both elevated CO_2_ (later: eCO_2_) and elevated O_3_ (later: eO_3_) have long been known to affect physiological and biochemical processes of plants and change rhizosphere conditions^27,28^. There is evidence that the composition and functioning of soil microbial communities change under eCO_2_^29,30^ and eO_3_^31,32^. A previous stuty showed that eCO_2_ increased soil bacterial abundance in soil contaminated with cadmium at various levels of concentration (0, 1.5, 3.0, 6.0 mg Cd kg^−1^soil) across the experimental period (2, 4, 6, 8 weeks)^29^, and eCO_2_ was reported to select for fungal communities that are more adapted to drought conditions^30^. Moreover, eO_3_ has been shown to eliminate the significant positive effect of eCO_2_ on cellobiohydrolase activity, but it did not alter the positive effect of eCO_2_ on N-acetylglucosaminidase activity^31^. Furthermore, eO_3_ substantially reduced the ectomycorrhiza colonization rate and ectomycorrhiza diversity in larch^32^. Tropospheric eCO_2_ has been shown to increase not only plant biomass, but also carbon inputs to soil, associated with higher soil microbial activity, and labile soil C representing elevated root exudation^33^. By contrast, eO_3_ decreases inputs of assimilates into the rhizosphere^32^, while both eCO_2_ and eO_3_ change the composition of root exudates released into the rhizosphere, thereby altering microbial biomass and activity in soils^34^. All these previous results suggest that plant diversity, eCO_2_, and eO_3_ may have strong interactive effects on ecosystem processes. As loss of plant diversity is likely to occur together with changes in tropospheric gas concentration, the present study for the first time tested their interactive effects on pollutant degradation, a central ecosystem function of soils^15^. Pollutant degradation is crucial as increasing anthropogenic activities pollute various ecosystems worldwide^35,36^.

Plant communities with different combinations of grasses, herbs, and legumes were set up in microcosms with PAHs-contaminated soil from a chemical plant in Nanjing, China. These microcosms were subjected to a full-factorial combination of ambient and predicted concentrations of CO_2_ and O_3_ in 2050^2^. After ten weeks of cultivation, PAH residuals in soils, soil microbial biomass and composition, soil enzymes, and plant biomass were determined. We expected (1) plant functional group richness to increase plant productivity^37^ and soil microbial biomass and activity^17,38^, (2) eCO_2_ to increase plant productivity and soil microbial biomass and activity^39,40^, (3) eO_3_ to decrease plant biomass and soil microbial biomass and activity^32^, and (4) the three global change drivers to interactively influence plant biomass, soil microbial functions, and the degradation of PAHs, e.g., with plant diversity amplifying the effect of eCO_2_^41^ or eCO_2_ buffering negative effects of eO_3_^42^.

## Results

### PAH residuals in soil

**Figure 1:**
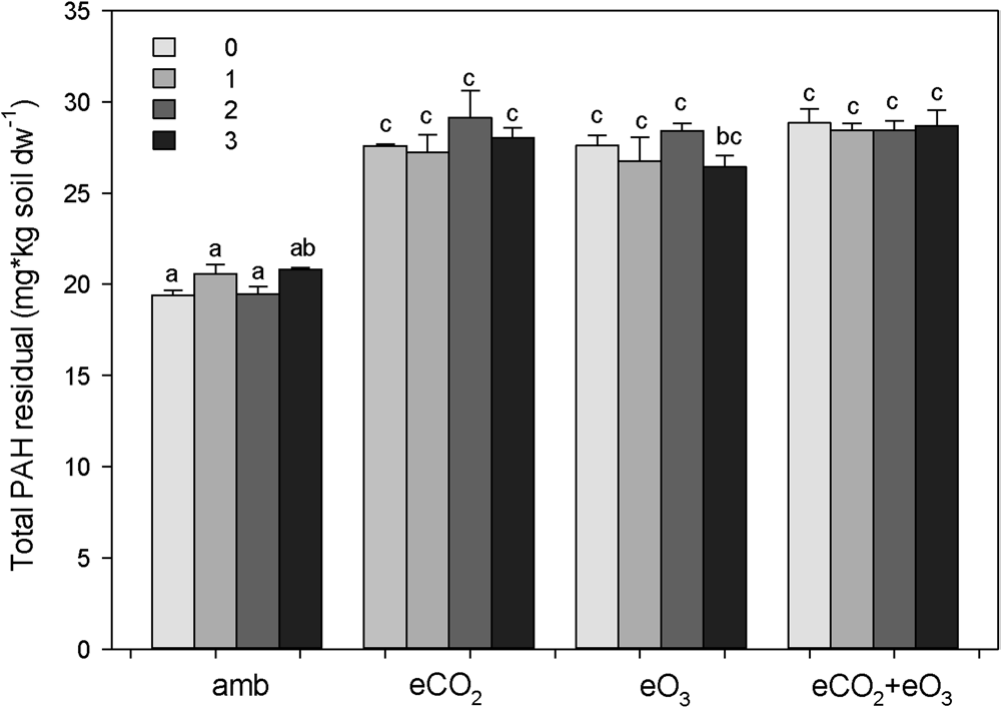
Total amount of polycyclic aromatic hydrocarbons (PAHs) after the experiment as affected by elevated CO_2_, elevated O_3_, and plant diversity (0, 1, 2, 3 plant functional groups). Means ± SE (n = 4). amb, eCO_2_, eO_3_, and eCO_2_ + eO_3_ means that microcosms were incubated in chambers with ambient air, with elevated CO_2_, with elevated O_3_, and with elevated CO_2_ and O_3_, respectively. Bars with different letters vary significantly (Tukey’s HSD test, a <0.05).

**Figure 2:**
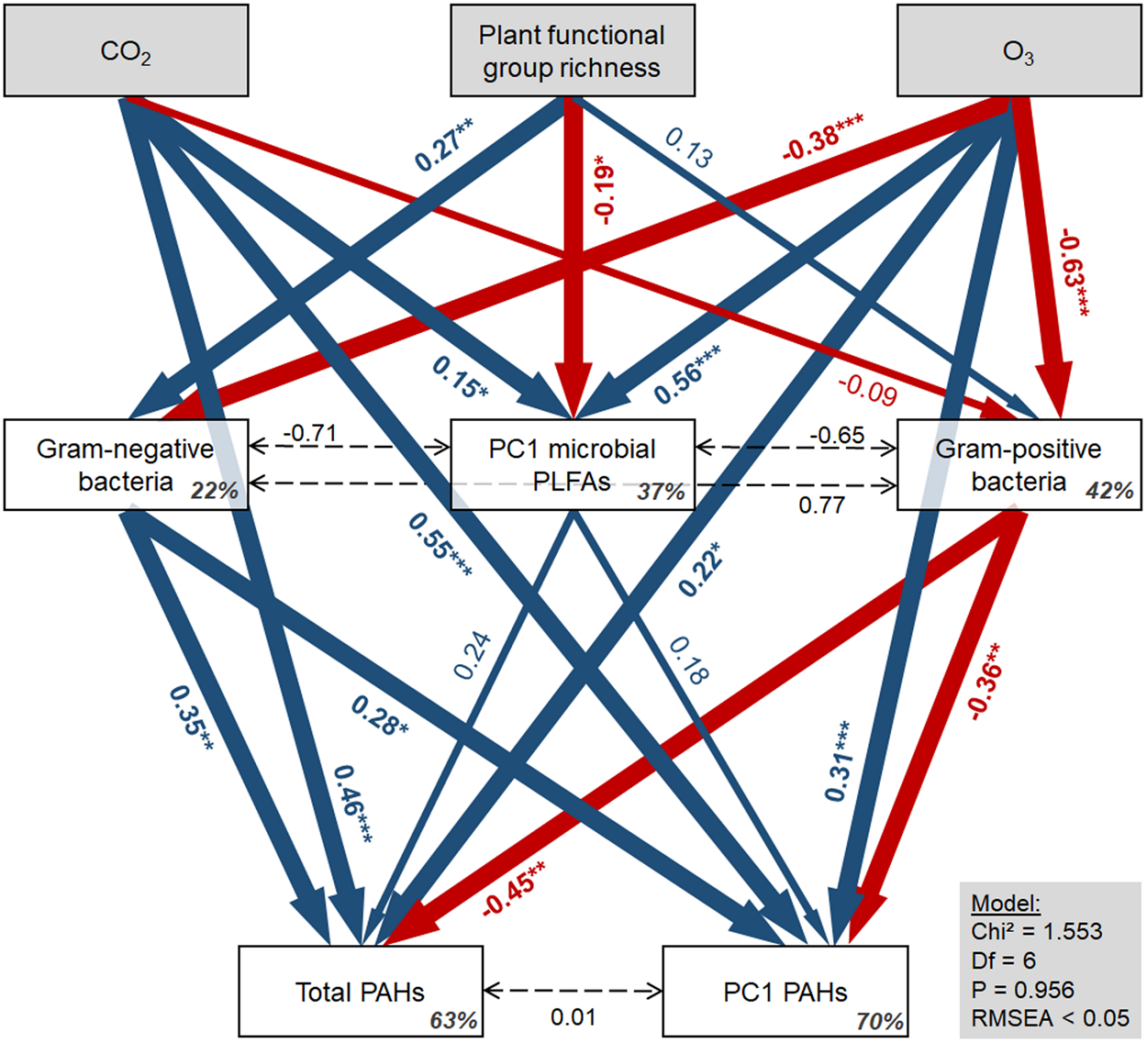
Structural equation model showing the effects of eCO_2_, eO_3_, and plant functional group richness on soil microorganisms and polycyclic aromatic hydrocarbon (PAHs) residuals in the soil. Red arrows: negative relationships, blue arrows: positive relationships, asterisks on numbers indicate significant relationships (see Table S1 for details).

**Figure 3:**
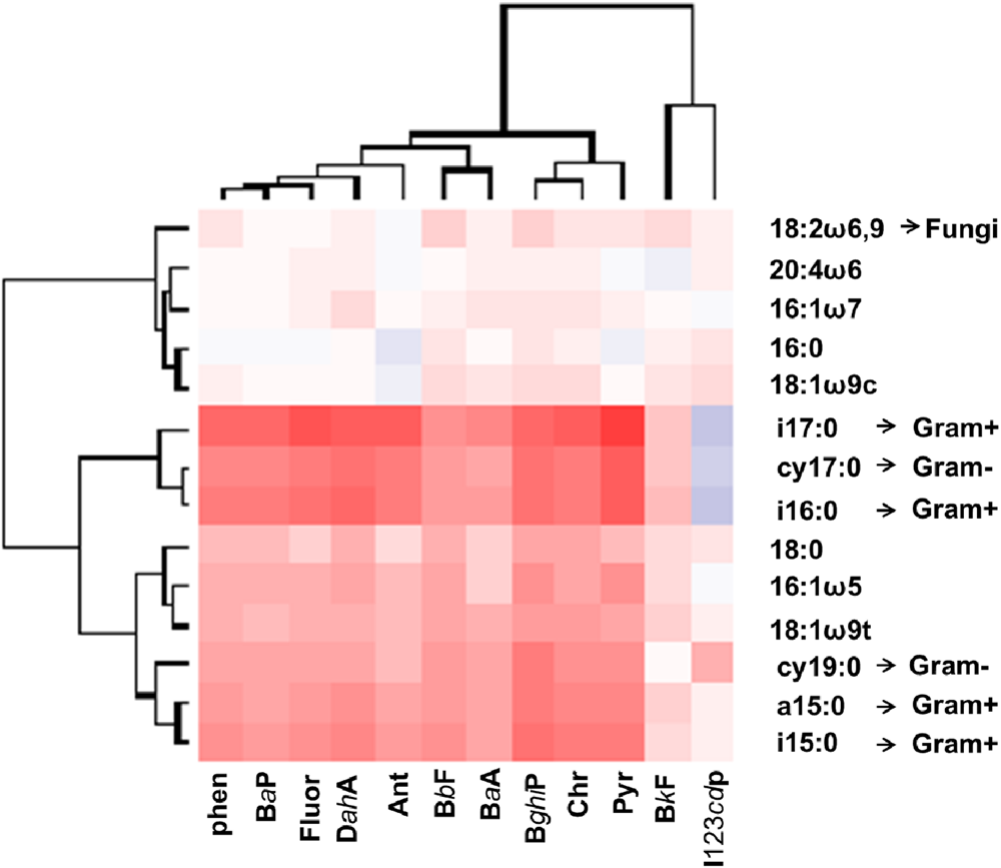
Heat map illustrating the relationships between different phospholipid fatty acids (PLFAs) and different polycyclic aromatic hydrocarbons (PAHs). Phen, BaP, Fluor, DahA, Ant, BbF, BaA, BghiP, Chr, Pyr, BkF, and I123cdP represent Phenanthrene, Benzo(a)pyrene, Fluoranthene, Dibenzo(a,h)anthracene, Anthracene, Benzo(b)fluoranthene, Benzo(a)anthracene, Benzo(g,h,i)perylene, Chrysene, Pyrene, Benzo(k)fluoranthene, and Indene(1,2,3-c,d)pyrene, respectively. Red plots: negative correlations, blue plots: positive correlations, white plots: no correlations.

Both eCO_2_ and eO_3_ increased total PAH residuals significantly (Table 1, Figs 1 and 2), *i.e*. decelerated PAH degradation, and also altered the composition of remaining PAHs (increased PC1 of PAHs; Fig. 2). CO_2_ × O_3_ had a significant interactive effect on total PAH residuals as remaining PAHs were lowest at aCO_2_ and aO_3_, but substantially increased by eCO_2_ and eO_3_ and highest at both eCO_2_ and eO_3_ (+43% in comparison to ambient conditions). Plant functional group richness had no significant effects on PAH residuals (Table 1). The PLFAs i17:0, cy 17:0, and i16:0 were most strongly associated with PAH removal, and Benzo(k)fluoranthene and Indene(1,2,3-c,d)pyrene were the most recalcitrant PAHs (Fig. 3). Gram-negative bacteria were positively associated with total PAH residuals and PC1 of PAH residuals, while Gram-positive bacteria strongly reduced total PAHs and PC1 of PAH residuals (Fig. 2).

**Table 1:** Analysis of variance results of total plant biomass, plant shoot biomass, plant root biomass, plant survival, biomass of gram-positive bacteria, biomass of gram-negative bacteria, biomass of fungi, phenol oxidase activity, polyphenol oxidase activity, and total PAHs residuals as affected by CO_2_, O_3_, plant functional group richness (FGR), and all interactions.

	CO_2_		O_3_		FGR		CO_2_ × O_3_		CO_2_ × FGR		O_3_ × FGR		CO_2_ × O_3_ × FGR		Error df
	F-value	P-value	F-value	P-value	F-value	P-value	F-value	P-value	F-value	P-value	F-value	P-value	F-value	P-value	
Total plant biomass	0.55	0.458	**5.55**	**0.020**	**3.30**	**0.041**	0.06	0.806	1.42	0.244	0.34	0.707	0.86	0.428	100
Plant shoot biomass	0.02	0.896	**6.39**	**0.013**	**4.18**	**0.018**	0.39	0.534	1.34	0.267	0.62	0.541	1.32	0.270	100
Plant root biomass	1.96	0.165	**4.09**	**0.046**	2.42	0.094	<0.01	0.934	1.27	0.285	0.18	0.836	0.44	0.642	100
Plant survival	<0.01	0.931	3.80	0.054	**4.88**	**0.010**	**4.27**	**0.041**	0.24	0.786	1.03	0.362	0.66	0.518	100
Gram-positive bacteria	**6.39**	**0.013**	**45.72**	<**0.001**	**7.92**	<**0.001**	**11.63**	**0.001**	**3.09**	**0.032**	2.12	0.104	1.06	0.371	82
Gram-negative bacteria	0.06	0.815	**7.72**	**0.007**	**11.82**	<**0.001**	1.92	0.169	2.17	0.097	0.91	0.441	0.53	0.666	82
Fungi	0.61	0.437	2.89	0.093	**3.37**	**0.022**	0.01	0.906	0.95	0.419	1.91	0.135	0.92	0.437	82
Phenol oxidase activity	1.94	0.167	0.21	0.646	**3.91**	**0.012**	**15.58**	<**0.001**	1.69	0.176	**2.77**	**0.047**	**2.92**	**0.039**	80
Polyphenoloxidase activity	**17.38**	<**0.001**	**70.11**	<**0.001**	**137.96**	<**0.001**	**22.18**	<**0.001**	**4.66**	**0.005**	1.92	0.133	0.32	0.814	80
Total PAHs	**79.37**	<**0.001**	**59.41**	<**0.001**	0.31	0.818	**42.95**	<**0.001**	0.13	0.944	0.45	0.718	1.84	0.147	79

P values in bold indicate significant effects (P < 0.05).

Although the fungal PLFA 18:2ω6, 9 was very abundant in the experimental soil (Fig. S3), it played a minor role in PAH degradation (Fig. 3). Furthermore, Gram-positive bacteria were the most important group of soil microbes in degrading PAHs (Figs 2 and 3).

### Soil enzymes and microorganisms

Both eCO_2_ and eO_3_ significantly reduced polyphenol oxidase activity, but plant functional group richness increased polyphenol oxidase activity (Table 1, Fig. S4b). However, enzyme activity depended on significant interactions between experimental factors (Table 1). CO_2_ × O_3_ had significant interactive effect on phenol oxidase and polyphenol oxidase activity. While the activity of phenol oxidase was lowest at aCO_2_ and aO_3_ and highest at eCO_2_ and aO_3_ (Fig. S4a), polyphenol oxidase activity was highest at aO_3_ and aCO_2_ or eCO_2_, but lowest at aCO_2_ and eO_3_ (Fig. S4b).

Furthermore, phenol oxidase activity was marginally affected by eO_3_ in the presence of 0 and 1 plant functional group, but increased phenol oxidase activity in the presence of 2 and 3 plant functional groups (significant O_3_ × plant functional group richness interaction). Phenol oxidase activity decreased with increasing plant functional group richness and was consistently higher at eCO_2_ and aO_3_, but did not vary with plant functional group richness and CO_2_ at eO_3_ (significant CO_2_ × O_3_ × plant functional group richness interaction). Moreover, the increase of polyphenol oxidase activity at eCO_2_ was most pronounced in the presence of two plant functional groups (significant CO_2_ × plant functional group richness interaction).

Elevated CO_2_ significantly reduced the biomass of Gram-positive bacteria (Table 1, Fig. S1a), and the composition of microbial communities changed as indicated by increased PC1 of microbial PLFAs (Table S1, Fig. 2). Furthermore, eO_3_ significantly reduced Gram-positive bacteria and Gram-negative bacteria (Tables 1, S1, Figs 2, S1a,b) and changed the composition of the soil microbial community (increased PC1 of microbial PLFAs; Table S1, Fig. 2). The biomass of Gram-positive bacteria, Gram-negative bacteria, and fungi increased significantly with increasing plant functional group richness (Table 1, Fig. S1a,b,c), which was also reflected by a significant change of soil microbial community composition (significantly reduced PC1 of microbial PLFAs; Table S1, Fig. 2). CO_2_ × O_3_ and CO_2_ × plant functional group richness had significant interactive effects on Gram-positive bacteria (Table 1). The biomass of Gram-positive bacteria was highest at aCO_2_ and aO_3_, lowest at aCO_2_ and eO_3_, and intermediate in the other treatments (Fig. S1a). Further, the biomass of Gram-positive bacteria increased with increasing plant functional group richness and was higher at aCO_2_, except in the treatment with two plant functional groups, where aCO_2_ and eCO_2_ had similar values.

The composition of microbial PLFAs corresponded to the functional composition of plant communities (Fig. S3). Plant communities containing legumes were more strongly associated with the PLFAs cy17:0, a15:0, 16:1ω7, i17:0, 18:0, i15:0, and cy19:0, almost all of them indicators of bacteria (except 16:1ω7 and 18:0). Plant communities containing herbs were more strongly associated with a different set of PLFAs, namely i16:0, 16:0, 18:1ω9t, 18:1ω9c, 20:4ω6, 16:1ω5, and 18:2ω6,9, with almost none of them being indicators of bacteria (except i16:0). Plant communities with grasses were associated with both bacterial indicator PLFAs and non-bacterial indicator PLFAs. (Fig. S3).

## Discussion

The present study shows that elevated CO_2_ and O_3_ concentrations may erode essential ecosystem services like the degradation of pollutants in soil by inducing significant shifts in soil microbial community structure and enzyme activity. These detrimental effects were consistent across plant communities differing in functional diversity. However, pronounced alterations of microbial community structure along the functional plant diversity gradient suggests that targeted and trait-based phytoremediation may help to counteract detrimental global change effects in long-term approaches.

In contrast to our hypothesis (1) stating that plant functional group richness to increase plant productivity^37^, plant biomass declined with increasing plant functional group diversity in the present study. These results highlight the context-dependency of biodiversity–ecosystem function relationships^43^ and the need to study biodiversity effects under stressful conditions^44^. Nevertheless and in line with our hypothesis^17,38^, soil microbial biomass and activity increased with plant functional group diversity, stressing the significance of plant diversity for soil functions. Diverse plant communities are expected to produce and release a higher quantity and diversity of organic compounds into their rhizosphere, which may sustain higher soil microbial biomass and activity^45^. Using long-term data from a grassland biodiversity experiment, Lange *et al*. found higher plant biodiversity to increase rhizosphere carbon inputs into the soil microbial community resulting in increased microbial diversity and activity^18^. These findings are consistent with the results of increased biomass of Gram-positive, Gram-negative bacteria and fungi in the present study. A recent meta-analysis reported that plant diversity effects on soil microbial biomass C were strong in long-term experiments and across various environmental contexts^46^. The present study extends those findings by showing that bacterial and fungal biomass increased with plant diversity, which also altered the activities of different soil enzymes. Similar with this study, Steinauer *et al*. found that soil microbial biomass and some enzyme activities increased with increasing plant diversity^47^.

In contrast to our hypotheses, no effect of plant functional group richness on PAH removal was observed. This is in line with a previous study that species richness had no significant effect on ^14^C-phenanthrene mineralization^48^. However, structural equation modeling (SEM) reveals a range of processes coupling plant functional group richness and PAH degradation. The SEM showed that plant diversity altered soil microbial community composition and favored both Gram-positive (accelerating PAH degradation) and Gram-negative bacteria (decelerating PAH degradation; Fig. 2). Plant community may therefore be an important driver of PAH degradation, even if lumping community composition into functional group richness doesn’t provide the adequate explanatory power. The present study suggests that it may be possible to assemble plant communities showing a high phytoremediation by steering soil microbial communities.

Elevated CO_2_ tended to increase plant productivity, although the results were only marginally significant. In addition, eCO_2_ had a negative impact on microbial processes linked to PAH degradation. Although eCO_2_ increased total soil microbial biomass and activity (Fig. 2, PC1 microbial PLFAs), it led to a decrease in Gram-positive bacteria, a microbial group linked to PAH degradation^49^ and the most important microbial group involved in the removal of PAHs from soil in the present study (Fig. 2). Furthermore, eCO_2_ altered the soil microbial community composition, which is also in line with previous studies^38,50,51^ and calls for more detailed investigations of shifts in soil microbial communities with sequencing techniques.

We propose that this effect of elevated tropospheric CO_2_ may be due to the higher plant carbon input in soil resulting from enhanced photosynthesis^52^. This may lead to higher soil microbial activity^29,53^, as the pool of labile soil C may be increased by elevated root exudation^33,54,55^. In the present study, eCO_2_ had non-significant effects on the biomass of fungi and Gram-negative bacteria, but decreased the biomass of Gram-positive bacteria (Fig. S1). Consistent with the present study, both Larson *et al*.^31^ and Grueter *et al*.^56^ found that eCO_2_ had no significant influence on microbial biomass and activity, while Manninen *et al*.^57^ found a negative effect of eCO_2_ on soil microbial biomass. These variable results indicate that eCO_2_ effects on soil microbial communities may depend on the environmental context, such as soil conditions and/or plant community composition^38^.

Elevated O_3_ decreased plant biomass, soil microbial biomass and activity, and PAH removal. Ozone is a toxic compound that can induce oxidative stress in plants, and high tropospheric O_3_ concentrations have been reported to decrease inputs and to change the composition of assimilates into the rhizosphere^34^, which in turns affects soil microbial communities. Results of the present study indicate that ozone-mediated changes in soil communities may have dramatic effects on soil self-cleaning potential. Consistent with past studies^58–60^, a strong decrease in the biomass of Gram-positive and Gram-negative bacteria and shifts in microbial community composition in response to eO_3_ was observed (Figs 2, S1).

The effects of eCO_2_, eO_3_, and plant diversity on PAH removal were mediated to some extent by alterations of soil enzymatic activity. Both elevated CO_2_ and O_3_ led to a decrease in polyphenol oxidase activity, while plant functional group richness increased polyphenol oxidase but decreased phenol oxidase. In line with the present study, eCO_2_ reduced the activity^61^ and abundance^62^ of polyphenol oxidase, suppressed phenol oxidase^63^, while enzymes including phenol oxidase were strongly affected by plant species richness^64^. These results indicate that simultaneous alteration of plant community composition and environmental conditions may have contrasting effects on enzyme activity involved in PAH removal. Notably, many enzymes are involved in the metabolism process of PAHs^65,66^, some of which were not measured here. Although the measured enzymes responded significantly to the treatments, this did not explain variation in PAH removal, which is why they were not considered in the structural equation model (Fig. 2).

Elevated CO_2_ and O_3_ concentrations and variations in plant diversity had significant interactive effects on plant biomass, soil microbial functions, and the degradation of PAHs. Plant diversity altered the effect of eCO_2_ on soil microbial biomass and activity, but the clear positive interaction effects as expected in hypothesis (4) were not detected. This highlights the importance of plant diversity and community composition in mediating soil microbial functions in a future world, but also calls for a better mechanistic understanding of interactive effects of plant diversity and global change drivers.

However, plant diversity did not alter eCO_2_ and eO_3_ effects on PAH removal in the present study. This is in line with a recent meta-analysis by Thakur *et al*.^46^ showing no interactive effects of plant diversity and global change factors in affecting soil microbial biomass in the short term. Potentially, plant diversity-induced differences in soil microbial community composition and subsequent effects on essential services like PAH degradation need a longer time than captured by the present experiment^46^. Moreover, we propose that lumping plant community composition into functional group richness may not provide the adequate explanatory level. Instead, we propose that future studies may use more targeted plant trait-based approaches^67^, e.g., by considering root/rhizosphere traits, to develop a better mechanistic understanding of the relationship between plant community composition and functioning of soil communities linked to pollutant removal.

Importantly and in contrast to our hypothesis (4), eCO_2_ amplified the inhibitory effect of eO_3_ on PAH removal. This effect was partly mediated by an enhancement of eO_3_ effects on most soil microbial groups at elevated CO_2_. It particularly amplified the negative effect of eO_3_ on Gram-positive bacteria, the most important microbial group driving the removal of PAHs from soil in this study. This result exemplifies how different global change drivers can have unexpected synergistic effects on soil functions and compromise important ecosystem services.

## Conclusion

We highlight that global environmental change factors, such as human-induced alterations in tropospheric gas composition, may undermine the ability of ecosystems to degrade pollutants. Soil self-cleaning showed a high robustness to alterations in plant diversity and community composition, yet elevated CO_2_ and O_3_ concentrations may compromise efforts such as phytoremediation to restore polluted soils. On the other hand, the present study also indicates that the targeted assembly of plant communities applying a more comprehensive knowledge regarding plant effects on soil biota may be a promising tool to shape soil microbial communities for the degradation of organic pollutants.

## Materials and Methods

### Open top chambers

The open top chamber (OTC) system is located at Xianlin campus, Nanjing University, Nanjing, China (118°57′36.15″E, 31°7′23.99″N). Briefly, this system consists of four chambers with full control of atmospheric CO_2_ and O_3_ concentrations: one chamber with ambient CO_2_ (aCO_2_) and ambient O_3_ (aO_3_) levels, one with eCO_2_ and aO_3_ levels, one with aCO_2_ and eO_3_ levels, and one with both eCO_2_ and eO_3_ levels. The glass chambers are octagonal with 2 m in diameter and 2.8 m in height. CO_2_ was released from a tank (Q/JB-THB002, Beijing Tianhai Industry Co., Ltd.), and O_3_ was produced by an O_3_ generator (NPF10/W, Shandong Lvbang Ozone Co., Ltd.) from pure O_2_. CO_2_ and/or O_3_ were mixed with air from temperature-controlled rooms and conveyed by fans (SFG-2, Shanghai Jiabao Co., Ltd.) to the bottom of the chambers. Gases were released into the antra *via* tiny holes in the stainless steel plate between the bottom and the antrum, and then released into the air of the open top of chambers. The quantity of the CO_2_ and O_3_ release was controlled by a flowmeter (LZB-3WB, Changzhou Shuangbo Co., Ltd.), the concentration of CO_2_ was detected with a CO_2_ monitor (Li-7000, Li-Cor, USA), and the concentration of O_3_ was detected with an O_3_ monitor (Model 205, 2B Co., USA). The O_3_ fumigation was conducted between 9:00 a.m. and 5:00 p.m. until harvest, except during rain events, and the CO_2_ fumigation was all day long until harvest. The target CO_2_ concentration for the eCO_2_ treatment was 200 ppm higher than aCO_2_, and the target O_3_ concentration for the eO_3_ treatment was 50–60 ppb higher than aO_3_ in order to simulate the forecasted tropospheric CO_2_ and O_3_ levels in 2050^2^.

### Plant cultivation

Three species of grasses (*Lolium perenne, Dactylis glomerata, Phleum pratense*), herbs (*Plantago lanceolata, Taraxacum officinale, Centaurea jacea*), and legumes (*Trifolium pratense, Trifolium repens, Medicago sativa*) were germinated in trays filled with quartz sand in the lab. Ten days after germination, seedlings were transplanted into the microcosms (8 cm in diameter and 12 cm in height) with 250 g of PAHs contaminated soil collected from a chemical plant in Nanjing (118°44′51.87″E, 31°58′4.71″N). Plant communities consisting of nine individuals and differing in functional group richness (8 different communities) were set up: bare ground (no plants); functional group ‘monocultures’ of either three grass species, three herb species, or three legume species; mixtures of two functional groups (grasses plus herbs, grasses plus legumes, or herbs plus legumes); and the mixture containing all three plant functional groups (grasses plus herbs plus legumes), thereby yielding functional group richness levels of 0, 1, 2, and 3 and functionally dissimilar plant communities. Each plant community was replicated four times per CO_2_ × O_3_ treatment (32 microcosms per OTC, 128 microcosms in total). Plant communities were cultivated in the lab for one week, and dead seedlings were replaced before microcosms were transferred to OTCs.

The microcosms were randomly placed in the OTCs, and each microcosm was watered with 10–20 ml of distilled water per day. After 10 weeks of cultivation in OTCs, plants and soils were sampled, survival of plants and plant community biomass was measured, and soils for PAHs determination were stored at −20 °C, whereas soils for the measurement of microbial parameters were stored at 4 °C.

### Determination of soil enzymatic activity

A very important step of PAH metabolism by bacteria and fungi is the breaking of PAH rings by phenol oxidase or polyphenol oxidase^65,66^. Therefore, these enzymes were used as proxy for general microbial processes linked to PAH degradation. For enzyme measurements, 0.5 g fresh soil was mixed with 20 ml milli-Q-water in 50 ml falcon tubes, shaken at 250 rpm for 30 min, centrifuged at 3000 rpm for 10 min, supernatants mixed with substrates and buffer in 96-well plates (Corning 96 Flat Bottom Transparent Polystyrol), then determined on a plate reader (Infinite M200, Tecan, Germany). Phenol oxidase activity was measured according to a modified protocol^68^. Briefly, 20 μl soil supernatant was mixed with 100 μl 5 mM bicarbonate buffer and 100 μl 5 mM L-3,4-dihydroxyphenylalanine (L-DOPA) solution, incubated at 27 °C, and absorbance was measured at 460 nm for 1 h. ΔA_460_/min from the initial linear portion of the curve was calculated. Polyphenol oxidase activity was measured according to Montgomery and Sgarbieri^69^. Briefly, 20 μl soil supernatants was mixed with 100 μl 0.5 M potassium phosphate buffer and 100 μl 1 mM 3-(4-hydroxyphenyl) alanine (L-Tyrosine) solution, incubated at 25 °C, and absorbance was measured at 280 nm for 12 min. ΔA_280_/min from the initial linear portion of the curve was calculated.

### PLFA analysis

PLFAs were extracted according to Bligh and Dyer^70^ modified by Kramer and Gleixner^71^. Briefly, soil lipids were extracted by a mixture of chloroform, methanol, and 0.05 M phosphate buffer (pH 7.4) and split up into phospholipids by eluting with chloroform, acetone, and methanol from a silica-filled solid phase extraction column. Subsequently, the phospholipids were hydrolyzed and methylated by a methanolic KOH solution, and the PLFA-methyl esters were identified and quantified by GC-ECD (PerkinElmer, Clarus 500, USA). PLFA 19:0 was used as internal standard. Separated phospholipid fatty acid methyl-esters were identified by chromatographic retention time and mass spectral comparison with a mixture of standard qualitative bacterial acid methyl-ester that ranged from C11 to C24 (Supelco). For each sample, the abundance of individual phospholipid fatty acid methyl-esters was expressed in nmol per g dry soil. The nomenclature for PLFAs followed that of Frostegård *et al*.^72^. The sum of PLFAs i14:0 i15:0, a15:0, i16:0, i17:0, and i18:0 represented the biomass of Gram-positive bacteria, that of PLFAs cy17:0 and cy19:0 represented the biomass of Gram-negative bacteria, and the amount of the fungal-specific fatty acid 18:2ω6,9 was used as an indicator of fungal biomass^73,74^.

### Determination of PAHs in soils

Soil samples stored at −20 °C were freeze-dried (Labconco 12 L, Labconco Co., USA) for 96 h, ground by mortars, and passed through a 2 mm sieve. Samples (5 g) were extracted with 20 mL methanol: methylene dichloride (1:2, v-v), concentrated in a rotary evaporator, and dried under a fine stream of nitrogen. The residues were dissolved in 0.5 ml acetonitrile. Samples were analyzed by high-performance liquid chromatography on a Spuelcosil^TM^ LC-PAH column (250 × 4.6 mm, 5 μm) (Supelco, Bellefonete, PA, USA) with UV detector at 254 nm (HPLC-UV, Hitachi L2000). The temperature of the column was kept constant at 30 °C to obtain reproducible retention times. The mobile phase consisted of water and acetonitrile in gradient mode at flow rate of 1 ml/min. The gradient solvent system started with 60% acetonitrile in water (v/v) during 10 min, then increasing linearly to 100% acetonitrile within 10 min, the 100% acetonitrile was maintained for 20 min, and finally returned to the initial conditions in 2 min.

### Statistical analyses

Analyses of variance (ANOVAs) were performed to test effects of CO_2_ (ambient and elevated), O_3_ (ambient and elevated), plant functional group richness (1, 2, 3 functional groups present), and all interactions on total plant biomass, plant shoot biomass, plant root biomass, plant survival, biomass of Gram-positive bacteria, biomass of Gram-negative bacteria, biomass of fungi, phenol oxidase activity, polyphenol oxidase activity, and total PAH residuals (for the latter, treatments with 0, 1, 2, and 3 functional groups were considered). If significant treatment effects were detected, additional Tukey’s HSD tests were performed to test for differences among means. ANOVAs were performed using Statistica 7.1 (Statsoft). Furthermore, structural equation modeling (SEM) was used to shed light on the mechanisms of PAH degradation by accounting for multiple potentially correlated effect pathways to disentangle the direct and indirect effects^75^ of experimental treatments and soil microbial community properties. The initial model was based on previous knowledge with experimental treatments as exogenous variables and the endogenous variables “Gram-negative bacteria”, “Gram-positive bacteria”, “PC1 microbial PLFAs” (representing PLFA composition), “PC1 PAHs” (representing PAH composition), and “total PAHs”. The adequacy of the models was determined *via* chi²-tests, AIC, and RMSEA^76^. Model modification indices and stepwise removal of non-significant relationships were used to improve the models; however, only scientifically sound relationships were considered^75^. Structural equation modeling was performed using Amos 5 (Amos Development Corporation, Crawfordville, FL, USA).

### Data availability

All data generated and/or analyzed during the current study are available from the corresponding author on reasonable request.

## Electronic supplementary material

Supplementary Information
