# Supplementary material for: Elevated tropospheric CO2 and O3 concentrations impair organic pollutant removal from grassland soil
Source: Sci Rep. 2018 Apr 3;8:5519. doi: 10.1038/s41598-018-23522-z (PMC5882802; doi:10.1038/s41598-018-23522-z)
Supplement: Supplementary file 1 — Supplementary Information [file 41598_2018_23522_MOESM1_ESM.pdf]

Elevated tropospheric CO_2 and O_3 concentrations impair organic pollutant removal from grassland soil

Fuxun Ai[1], Nico Eisenhauer[2,3], Alexandre Jousset[4], Olaf Butenschoen[5], Rong Ji[1], Hongyan Guo[1*]

[1]*Stake Key Laboratory of Pollution Control and Resource Reuse, School of Environment, Nanjing University, Nanjing 210023, China*

[2] *German Centre for Integrative Biodiversity Research (iDiv) Halle-Jena-Leipzig, Deutscher Platz 5e, 04103 Leipzig, Germany*

[3]*Institute of Biology, Leipzig University, Deutscher Platz 5e, 04103 Leipzig, Germany*

[4]*Institue of Environmental Biology, Utrecht University, Padualaan 8, 3584 CH, Utrecht, The Netherlands*

[5]*J.F. Blumenbach Institute of Zoology and Anthropology, University of Göttingen, Berliner Str. 28, 37073, Göttingen, Germany*

*Corresponding author

Mobile phone: +86 13813945566

Email address: hyguo@nju.edu.cn

Table S1. Results of structural equation modeling of the effects of elevated CO_2, elevated O_3, and plant functional group richness on the biomass of Gram-negative bacteria (GramNEG), Gram-positive bacteria (GramPOS), soil microbial community composition (PC1 PLFA), composition of remaining polycyclic aromatic hydrocarbons (PAHs), and total amount of PAHs in soils (see Fig. 2).

Variables			*Estimate*	*S.E.*	*C.R.*	*P*
**GramNEG**	**←**	**O_3**	**-127.901**	**34.464**	**-3.711**	**<0.001**
**GramNEG**	**←**	**FunctRich**	**66.181**	**24.620**	**2.688**	**0.007**
**GramPOS**	**←**	**O_3**	**-203.392**	**28.468**	**-7.145**	**<0.001**
GramPOS	←	FunctRich	31.330	20.337	1.541	0.123
GramPOS	←	CO_2	-29.889	18.216	-1.641	0.101
**PC1 PLFAs**	**←**	**CO_2**	**1.033**	**0.446**	**2.314**	**0.021**
**PC1 PLFAs**	**←**	**FunctRich**	**-0.925**	**0.451**	**-2.052**	**0.040**
**PC1 PLFAs**	**←**	**O_3**	**3.921**	**0.631**	**6.211**	**<0.001**
**Total PAHs**	**←**	**GramNEG**	**0.000**	**0.000**	**2.663**	**0.008**
**PC1 PAHs**	**←**	**GramNEG**	**0.001**	**0.000**	**2.305**	**0.021**
**PC1 PAHs**	**←**	**O_3**	**0.277**	**0.078**	**3.570**	**<0.001**
**PC1 PAHs**	**←**	**CO_2**	**0.485**	**0.058**	**8.340**	**<0.001**
**PC1 PAHs**	**←**	**GramPOS**	**-0.001**	**0.000**	**-2.700**	**0.007**
**Total PAHs**	**←**	**GramPOS**	**0.000**	**0.000**	**-3.095**	**0.002**
**Total PAHs**	**←**	**O_3**	**0.032**	**0.014**	**2.235**	**0.025**
**Total PAHs**	**←**	**CO_2**	**0.068**	**0.011**	**6.365**	**<0.001**
Total PAHs	←	PC1 PLFAs	0.005	0.003	1.871	0.061
PC1 PAHs	←	PC1 PLFAs	0.022	0.015	1.521	0.128

Given are the unstandardized path coefficients (estimates), standard error of regression weight (S.E.), the critical value for regression weight (C.R.; z = estimate / S.E.) and level of significance for regression weight (P). Significant effects are highlighted in bold font.

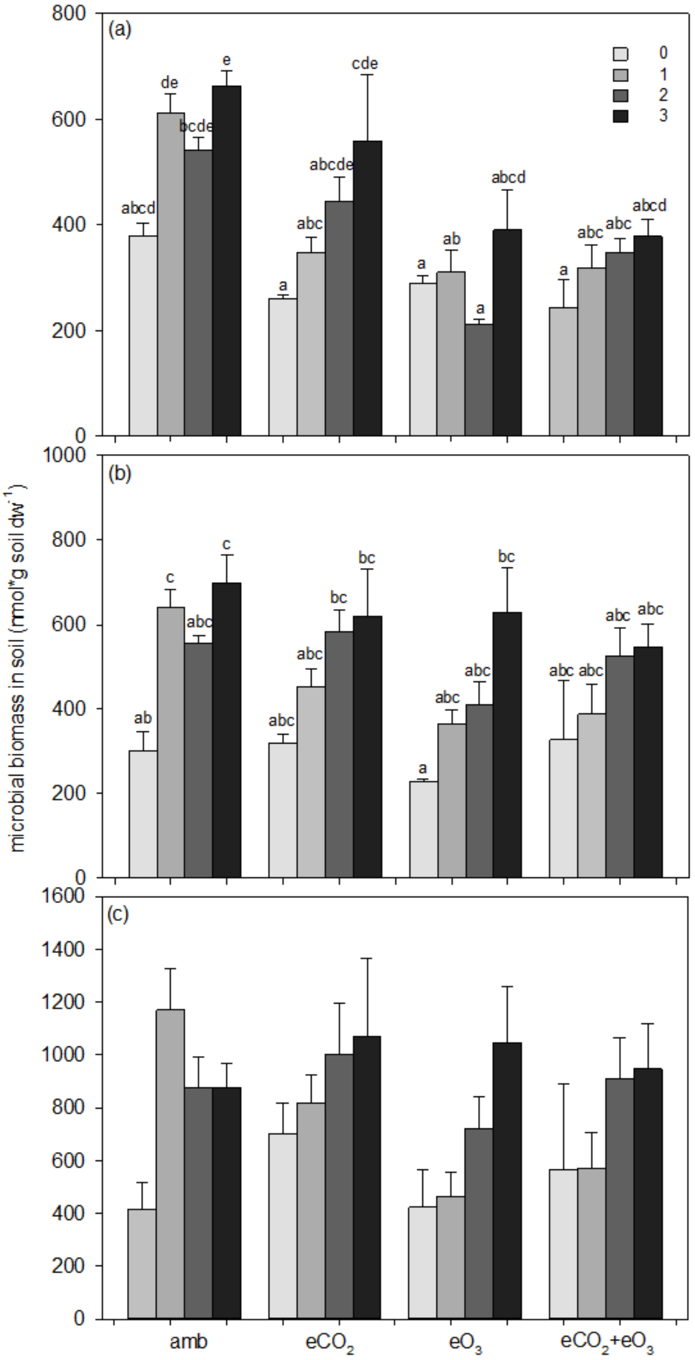

Figure S1. Biomass of Gram-positive bacteria (a), Gram-negative bacteria (b), and fungi (c) as affected by elevated CO_2, elevated O_3, and plant diversity (0, 1, 2, 3 plant functional groups). Means ± SE (n = 4). amb, eCO_2, eO_3, and eCO_2+eO_3 means that microcosms were incubated in chambers with ambient air, with elevated CO_2, with elevated O_3, and with elevated CO_2 and O_3, respectively. Bars with different letters vary significantly (Tukey's HSD test, a<0.05).

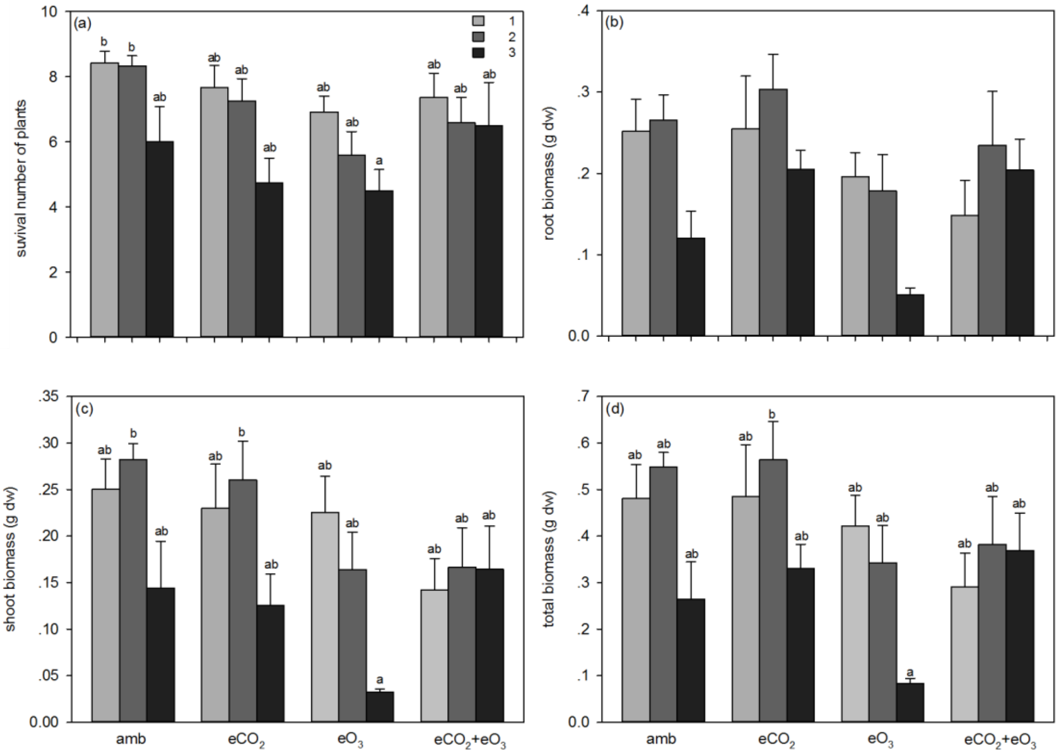

Figure S2. Plant survival (a), root biomass (b), shoot biomass (c), and total plant biomass (d) as affected by elevated CO_2, elevated O_3, and plant diversity (1, 2, 3 plant functional groups). Means ± SE (n = 4). amb, eCO_2, eO_3, and eCO_2+eO_3 means that microcosms were incubated in chambers with ambient air, with elevated CO_2, with elevated O_3, and with elevated CO_2 and O_3, respectively. Bars with different letters vary significantly (Tukey's HSD test, a<0.05).

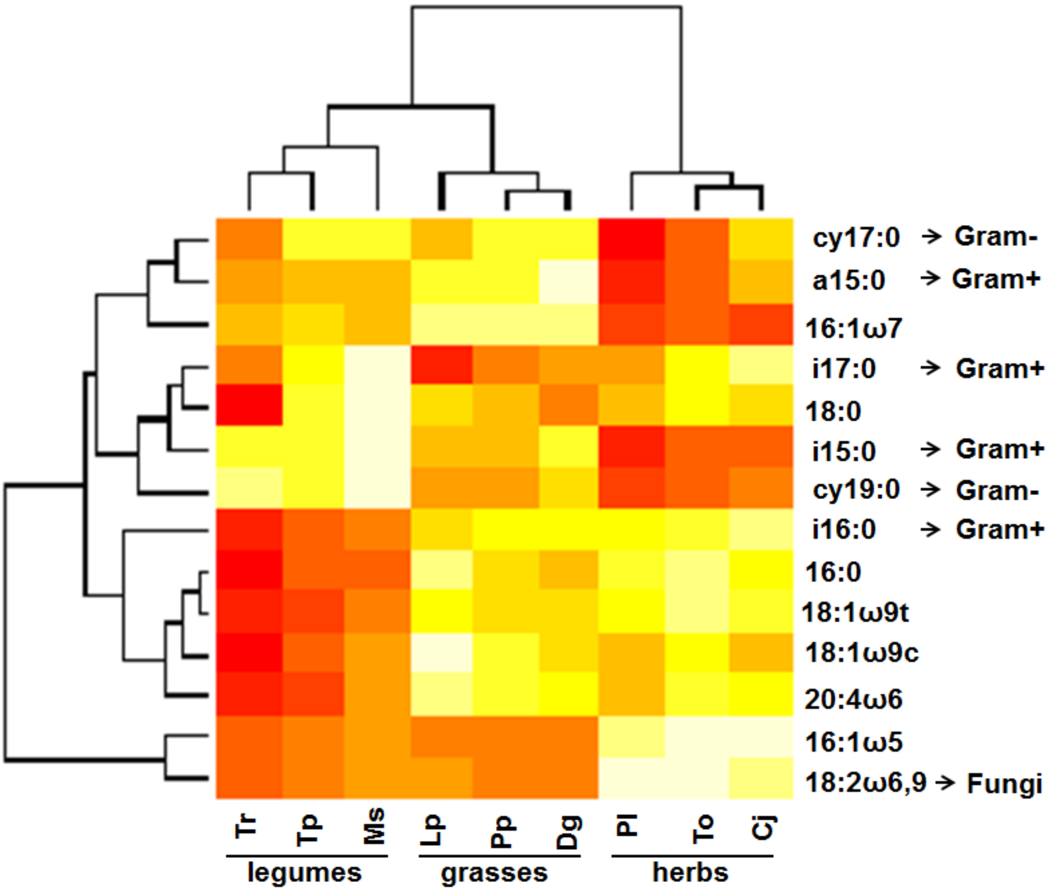

Figure S3. Heat map illustrating the relationships between different phospholipid fatty acids (PLFAs) and different plant species. Tr, Tp, Ms, Lp, Pp, Dg, Pl, To, Cj represent *Trifolium repens, Trifolium pratense, Medicago sativa*, *Lolium perenne*, *Phleum pretense*, *Dactylis glomerata, Plantago lanceolata*, *Taraxacum officinale*, and *Centaurea jacea*, respectively. Red plots: negative correlations, yellow plots: positive correlations, white plots: no correlations.

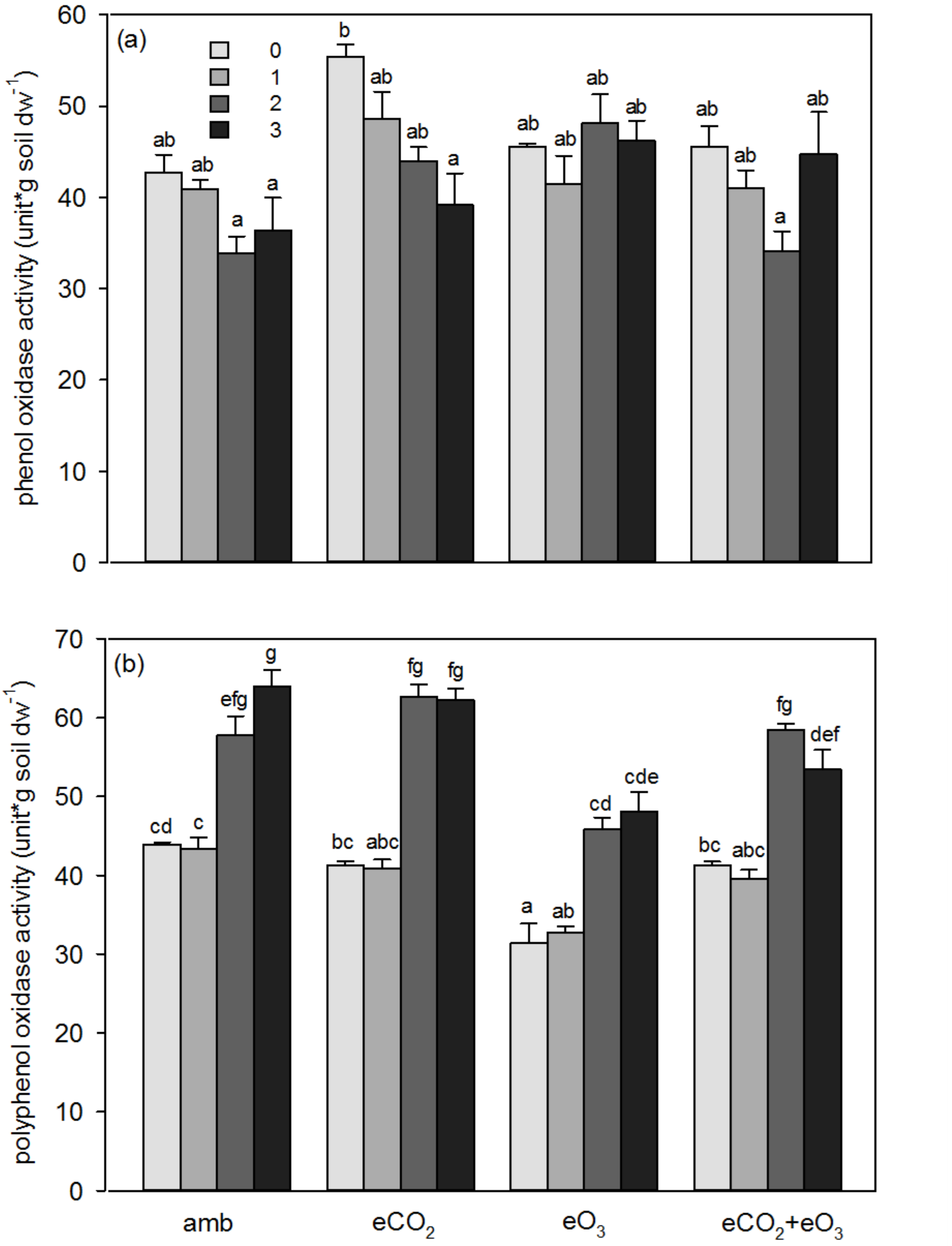

Figure S4. Phenol oxidase activity (a) and polyphenol oxidase activity (b) as affected by elevated CO_2, elevated O_3, and plant diversity (0, 1, 2, 3 plant functional groups). Means ± SE (n = 4). amb, eCO_2, eO_3, and eCO_2+eO_3 means that microcosms were incubated in chambers with ambient air, with elevated CO_2, with elevated O_3, and with elevated CO_2 and O_3, respectively. Bars with different letters vary significantly (Tukey's HSD test, a<0.05).
